# Identification of Natural Compounds of the Apple as Inhibitors against Cholinesterase for the Treatment of Alzheimer’s Disease: An In Silico Molecular Docking Simulation and ADMET Study

**DOI:** 10.3390/nu15071579

**Published:** 2023-03-24

**Authors:** Qazi Mohammad Sajid Jamal, Mohammad Imran Khan, Ali H. Alharbi, Varish Ahmad, Brijesh Singh Yadav

**Affiliations:** 1 Department of Health Informatics, College of Public Health and Health Informatics, Qassim University, Al Bukayriyah 52741, Saudi Arabia; m.quazi@qu.edu.sa (Q.M.S.J.); ahhrbie@qu.edu.sa (A.H.A.); 2 Department of Biochemistry, Faculty of Science, King Abdulaziz University, Jeddah 21589, Saudi Arabia; mikhan@kau.edu.sa; 3 Centre for Artificial Intelligence in Precision Medicines, King Abdulaziz University, Jeddah 21589, Saudi Arabia; vaahmad@kau.edu.sa; 4 Health Information Technology Department, The Applied College, King Abdulaziz University, Jeddah 21589, Saudi Arabia; 5 Faculty of Biosciences and Aquaculture, Nord University, N-8026 Bodø, Norway

**Keywords:** Alzheimer’s disease, apple flavonoids, acetylcholinesterase, butyrylcholinesterase, molecular docking

## Abstract

Alzheimer’s disease (AD), the most common type of dementia in older people, causes neurological problems associated with memory and thinking. The key enzymes involved in Alzheimer’s disease pathways are acetylcholinesterase (AChE) and butyrylcholinesterase (BuChE). Because of this, there is a lot of interest in finding new AChE inhibitors. Among compounds that are not alkaloids, flavonoids have stood out as good candidates. The apple fruit, *Malus domestica* (Rosaceae), is second only to cranberries regarding total phenolic compound concentration. Computational tools and biological databases were used to investigate enzymes and natural compounds. Molecular docking techniques were used to analyze the interactions of natural compounds of the apple with enzymes involved in the central nervous system (CNS), acetylcholinesterase, and butyrylcholinesterase, followed by binding affinity calculations using the AutoDock tool. The molecular docking results revealed that CID: 107905 exhibited the best interactions with AChE, with a binding affinity of −12.2 kcal/mol, and CID: 163103561 showed the highest binding affinity with BuChE, i.e., −11.2 kcal/mol. Importantly, it was observed that amino acid residue Trp286 of AChE was involved in hydrogen bond formation, Van Der Walls interactions, and Pi–Sigma/Pi–Pi interactions in the studied complexes. Moreover, the results of the Molecular Dynamics Simulation (MDS) analysis indicated interaction stability. This study shows that CID: 12000657 could be used as an AChE inhibitor and CID: 135398658 as a BuChE inhibitor to treat Alzheimer’s disease and other neurological disorders.

## 1. Introduction

Alzheimer’s disease (AD), the most common form of dementia in older people, is a significant cause of disability today and is linked to impairments in memory and thinking. There is currently no treatment or cure for Alzheimer’s disease (AD) due to its complex biochemical process [1,2]. Two neurochemical changes in Alzheimer’s disease are cholinergic deficit and a decreased synthesis of choline, which cause the abnormal activities of some the enzymes involved in neurological signaling [3,4]. Acetylcholinesterase (AChE) and butyrylcholinesterase (BuChE) are the two main cholinesterases (ChEs) found in the brain, detected in neurofibrillary tangles and neuritic plaques. AChE and BuChE are hydrolytic enzymes that cleave acetylcholine (ACh) into choline and acetate, thereby terminating synaptic cleft functioning. Previously presented scientific reports have stated that the healthy brain is enriched in significant levels of AChE and BuChE, which play a minor role in regulating brain ACh levels. But in people with Alzheimer’s, BuChE activity slowly increases while AChE activity stays the same or goes down. As a result, both enzymes are promising therapeutic targets for improving the cholinergic deficit that is thought to be behind the declines in cognitive, global functioning, and behavioral status that comes with AD [5]. Even though the cause of AD is unknown, previously conducted research indicated that the activity of cholinesterase (ChE) needs to be controlled at various key points of AD pathogenesis. One of the most effective treatment strategies has been to inhibit AChE and BuChE, which inhibited cholinergic function and increased ACh levels. AChE and BuChE inhibitors have been developed and used to treat Alzheimer’s disease by boosting cholinergic neurotransmitter activity in the brain, thereby reducing AD symptoms [6,7]. The way cholinesterase inhibitors exert their mechanism of action under the cholinergic hypothesis dictates that AD is linked to a loss of cholinergic function in the central nervous system (CNS). An aging brain’s loss of cholinergic function is associated with a gradual decline in neuronal function [8,9].

This loss of cholinergic activity may be related to or linked to several things, such as the production of amyloid peptide and the clumping together of tau protein, among others [10], stress [11], and excessive transition metals [12]. Surprisingly, AChE inhibitors have been shown to influence the “amyloid cascade” [13], which starts with the aggregation of insoluble amyloid β in the brain [14]. But AChE may also make A-peptides, which would speed up the process. It looks like the enzyme’s peripheral anionic site (PAS) is very important for this activity [15,16].

To date, three of the four medications for treating Alzheimer’s disease that have been authorized were from AChE inhibitor development programs [17]. Tacrine (1, 2, 3, 4-tetrahydro-9-aminoacridine) has been used to treat Alzheimer’s dementia. It was the first ChE inhibitor to receive FDA approval, and many different AChE inhibitors, like galantamine, donepezil, rivastigmine, etc., were created in the years that followed [18,19]. Many plant extracts have been investigated for their potential to treat neurological and cognitive problems. Galantamine was the first plant-derived AChE inhibitor discovered [20]. Many herbal remedies, including olive, tea, blueberry, strawberry, peppermint, walnut, immortelle, and sage, have been documented to have AChE-inhibiting activities due to the presence of polyphenols [21,22,23,24]. Curcumin, (-)-epigallocatechin-3-gallate (EGCG), and several flavonoids were also effective AChE inhibitors when isolated [25,26,27]. Bisphenols possess structure-specific inhibitory activity, and they can block either acetylcholinesterase (AChE) or butyrylcholinesterase (BuChE) [28,29,30]. While caffeic and quinic acids did not inhibit either AChE or BuChE, chlorogenic acid and 3-O-caffeoylquinic acid did, according to Chan et al. [31,32]. This is a polyphenolic compound found in apples (quercetin) that may aid in the prevention of Alzheimer’s disease. Turmeric curcumin, green tea’s main active phenolic compound, EGCG, and resveratrol have all been linked to AChE inhibition [33,34,35,36]. The flavanone naringenin, a major flavonoid in citrus, has been shown to exert AChE inhibitory activity in vitro and anti-amnesic activity in vivo [37,38]. Although the flavonol quercetin’s inhibitory effect has not been examined in vivo, it also seems to affect cholinergic dysfunction and cerebral blood flow in the brain [39]. AChE inhibitors should be developed for a variety of reasons. Existing drugs (donepezil, galantamine, and rivastigmine) have limitations in terms of efficacy and tolerability, and Alzheimer’s disease is characterized by complex and multifactorial pathological mechanisms involving multiple neurotransmitter systems, inflammation, oxidative stress, and abnormal protein accumulation [40]. Therefore, the creation of novel AChE inhibitors is of interest, and potential candidates have been found among non-alkaloid substances, such as flavonoids.

The apple, scientifically known as *Malus domestica* (Rosaceae), comes in at number two on the list of foods with the highest total concentration of phenolic compounds, behind only cranberries. Apples contain five different polyphenols, including flavanols, phenolic acids, dihydrochalcones, and anthocyanins [41]. Phytochemicals, particularly flavonoids, can be found in apples in varying concentrations depending on factors such as growing conditions, harvesting time, and how the fruit is processed. Flavonoids vary in type and concentration depending on whether they are found in the apple bark or core.

Flavonoids found in apples include the well-known quercetins (quercetin-3-galactoside, quercetin-3-glucoside, and quercetin-3-ramnoside), as well as other compounds like epicatechin catechin, cyanidin-3-galactoside, procyanidin, chlorogenic acid, coumaric acid, phlorizin, and gallic acid [42]. Several different types of flavonoid conjugates, including procyanidins, catechins, epicatechins, chlorogenic acid, fluorine, and quercetin, can be found in apple bark. The phytonutrients catechol, epicatechin, procyanidin, and fluorine are present in the apple core but in significantly lower concentrations than in the apple bark [43]. As a result, the quercetin conjugates are only found in the bark, and chlorogenic acid is the only flavonoid more abundant in the apple core than in the bark [44]. Apples have been the subject of much research because of their possible health benefits, including protecting against and treating chronic diseases like Alzheimer’s. Scientific evidence suggests that the high flavonoid content of apple juice and concentrate may help reduce the symptoms of Alzheimer’s disease, laying the groundwork for future controlled clinical trials [45,46]. In rodent models, apple extracts high in anthocyanins and flavan-3-ols have been shown to slow the progression of Alzheimer’s disease. This adds to the growing body of evidence supporting the use of polyphenols for cognitive health in the elderly [47]. An iron- and folate-deficient diet in adults and aged mice causes acetylcholine levels to drop, demonstrating that eating antioxidant-rich foods like apples can prevent the decline in cognitive performance associated with dietary and genetic deficiencies and aging. Apple juice concentrate added to drinking water has the same effect [27]. Caffeic acid also reduced acetylcholinesterase activity and nitrite production significantly. It also decreased inflammation, oxidative stress, nuclear factor-B-p65 protein expression and activity, and p53, caspase-3, and phosphorylated (p-)p38 MAPK activity [48].

Exploration of novel or alternative cheap molecules from natural resources is always in demand, and research continues. One of the faster and most cost-effective techniques is computational techniques. Thus, using computational biology, it has been observed that various classes of chemicals from plants and marine origins have been screened and reported to have significant inhibitory activity against cholinesterase. Still, cholinesterase inhibitors from fruits are not explored. Thus, in this study, we conducted a virtual screening to find novel cholinesterase inhibitors from fruits and reported the molecular conformations of apple chemicals that interact with cholinesterase. Docking and molecular simulation tools were used to learn more about the importance of binding interactions of potentially novel molecules for the treatment of AD. Therefore, apple consumption by the AD patient could be significant in managing AD.

## 2. Material and Methods

In search of AChE and BuChE inhibitors from a library of the apple’s natural compounds, we have adopted molecular docking-based virtual screening between natural compounds and selected enzymes. The required data was downloaded from structural databases like Protein Data Bank (PDB) (www.rcsb.org) (accessed on 23 December 2022) [49] and PubChem (https://pubchem.ncbi.nlm.nih.gov) (accessed on 23 December 2022) [50]. Online tools were used to perform ADMET profiling of the identified natural compounds. The 2D and 3D graphics were developed using Discovery Studio visualizer 2021 [51]. The obtained docking data was last validated by Molecular Dynamics Simulation (MDS) methods. We have provided details of each technique in the following sections.

### 2.1. Preparation of Ligand Structures

*Malus domestica*’s 164 natural compounds library in structure-data file (.sdf) format was mined and downloaded from the PubChem database. The drug rivastigmine was taken as a control molecule, and its chemical file was retrieved from the DrugBank Database (https://go.drugbank.com/drugs/DB00989 (accessed on 23 December 2022)) [52].

### 2.2. Preparation of Enzyme Structures

We have downloaded the 3D structure of human AChE (PDB:7E3H) developed by X-ray diffraction with a resolution of 2.45 Å, R-Value free of 0.224, R-Value work of 0.194, and R-Value observed of 0.195; while BuChE (PDB:7AIY) was prepared by X-ray diffraction with a resolution of 2.94 Å, R-Value free of 0.300, R-Value work of 0.225, and R-Value observed of 0.229. Initially, from the native 3D structures of the selected enzymes, HETATM and water molecules were removed after .pdb file editing in Discovery Studio Visualizer 2021 [51]. Then, CHARMm forcefield [53] was used to perform energy minimization of the selected receptors [51].

### 2.3. Virtual Screening

The fast virtual screening was performed using the PyRx tool after uploading the natural compounds and receptor molecules in the execution tool [54].

### 2.4. Molecular Interaction Analysis

Binding affinity between the apple’s natural compounds and the enzymes was calculated after docking analysis with AutoDock suite [55], which is built in PyRx. The AutoDock tool uses the scoring function of the chemical compound and protein molecules interaction according to the binding energy (Δ*G*) calculation based on the following formula:

Δ*G*_binding_ = Δ*G*_gauss_ + Δ*G*_repulsion_ + Δ*G*_hbond_ + Δ*G*_hydrophobic_ + Δ*G*_tors_,

where Δ*G*_gauss_: attractive term for dispersion of two gaussian functions; Δ*G*_repulsion_: square of the distance if closer than a threshold value; Δ*G*_hbond_: ramp function—also used for interactions with metal ions; Δ*G*_hydrophobic_: ramp function; Δ*G*_tors_: proportional to the number of rotatable bonds [56].

The molecular docking-assisted virtual screening was executed on the active site after setting the grid box to 25 × 25 × 25 Å, which covered key amino acid residues of the active site. Default molecular docking parameters were utilized for obtaining the best conformation of the apple’s natural compounds and AChE/BuChE complexes. The 3D models of complexes containing hydrogen bond information, residues involved in hydrogen bonding, Van Der Waals interactions, and Pi–Pi/Pi–alkyl bonds were obtained from Discovery Studio Visualizer 2021 [51,56,57].

### 2.5. Drug-Likeness and ADMET

*In silico* pharmacokinetics properties and drug-likeness predictions of absorption, distribution, metabolism, and excretion (ADME) of the selected natural compounds were performed using the SwissADME server developed by the Swiss Institute of Bioinformatics (SIB) [58,59,60]. Also, additional toxicity analysis prediction was made using the pkCSM tool [61].

### 2.6. Molecular Dynamics Simulation

We performed MDS of the natural compounds that best interacted with the AChE and BuChE enzymes. A 50 nanoseconds (ns) simulation was executed for each complex with the Groningen Machine for Chemical Simulations (GROMACS) 2021 tool. The GROMACS standard protocol was followed and other required methodologies were adopted from our previously published articles [59,60,62]. The pdb2gmx module was used to generate AChE and BuChE topology files, and then the CHARMM27 all-atom force field was chosen for simulation. The SwissParam server was then used to generate the natural compounds topology files [63]. A solvation unit cell box filled with water molecules was prepared in a triclinic shape. The new box volume was 241.76 (nm^3^) with a system size of 6.260 5.173 7.464 (nm), from center −5.437 3.284 −2.865 (nm), box vectors 6.261 5.173 7.464 (nm), and the box angles were 90.00 90.00 90.00 (degrees). The ligand–protein complexes were created and solvated in water for the cellular model. In order to neutralize the system, Na^+^ or Cl^−^ ions were utilized, followed by energy minimization. Initially, the system containing each complex had to be set up in equilibrium, and then two different ensembles—the NVT (constant number of particles, pressure, and temperature) ensemble and the NPT (constant number of particles, pressure, and temperature) ensemble were carried out. Both ensembles provide control over temperature and pressure coupling, resulting in constancy and stabilization of the system through complete simulation. We used gmx rms for root mean square deviation (RMSD) [64], gmxrmsf for root mean square fluctuation (RMSF), gmx gyrate for the radius of gyration (Rg) [65], and gmxhbond for the calculation of the number of hydrogen bonds made between compounds and enzymes. Trajectory files were generated and required simulation plots were created using the Xmgrace program as described by Turner, 2005 [66].

## 3. Results and Discussions

### 3.1. Docking Results

The *in silico* results obtained by docking analysis are documented in Table 1 and Table 2. The molecular docking results reveal that the selected natural compounds of the apple exhibited interactions with acetylcholinesterase (AChE) and butyrylcholinesterase (BuChE) in their active pockets compared to the chosen control drug rivastigmine. The preferred drug is a parasympathomimetic or cholinergic compound from the cholinesterase inhibitor class, working as a dual inhibitor against AChE and BuChE. This drug has U.S. Food and Drug Administration (FDA)-approved status for treating Alzheimer’s disease and other neurological disorders. All kinds of compounds were found to bind easily in the same area with a slight deviation (Supplementary File Figure S1).

**Figure 1:**
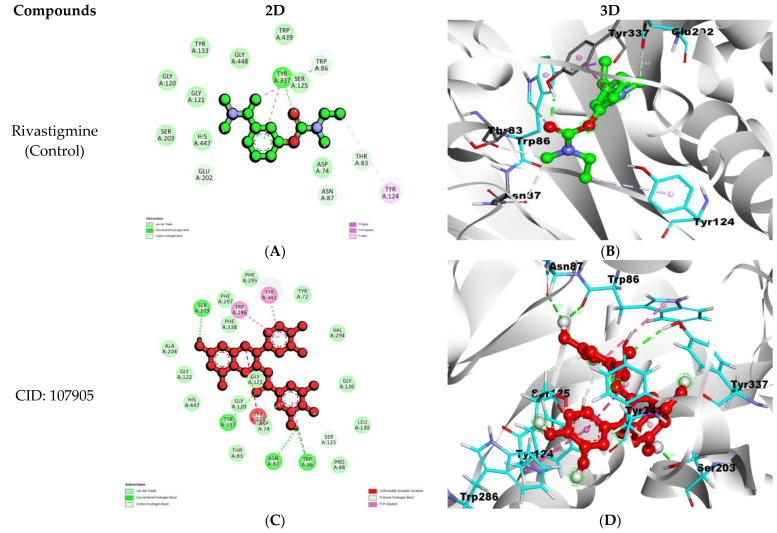

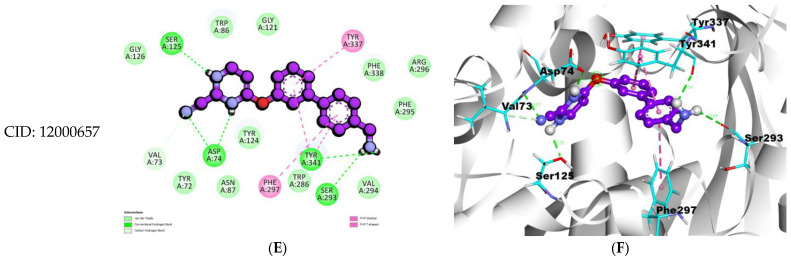
2D representations (**A**,**C**,**E**) and 3D conformations (**B**,**D**,**F**) of the interactions of the selected natural compounds with AChE.

CID: 107905 interaction with AChE formed a total of six hydrogen bonds, and amino acid residues Ala204, Gly122, His447, Thr83, Asp74, Gly120, Gly121, Pro88, Leu130, Gly126, Val294, Tyr72, Phe295, Phe297, and Phe338 were involved in Van Der Waals interactions, while Trp286 and Tyr341 were forming Pi–Pi interactions (Table 1; Figure 1C,D). Further, the CID: 12000657 with AChE complex formed a total of six hydrogen bonds. Amino acid residues, namely Tyr72, Val73, Trp86, Asn87, Tyr124, Gly121, Ser125, Gly126, Trp286, Val294, Phe295, Arg296, and Phe338 were involved in Van Der Waals interactions. Other interaction types also formed Phe297, Tyr337, and Tyr341 were involved in Pi–Pi interactions (Table 1; Figure 1E,F).

AChE and BuChE have four types of pockets: acyl, catalytic triad, choline-binding, and peripheral anionic pockets. Both enzymes hydrolyzed at the active site situated 20 Å deep inside the pocket. The amnio acid residue Asp74 of AChE is responsible for ligand binding, well supported by Trp286, while Asp70 of BuChE is present in the peripheral anionic pocket and plays a significant role in ligand interaction. Also, some aryl residues like Phe295 and Phe297 of AChE, and Phe329 and Tpr332 of BuChE, pull ligands toward the inner gorge [67,68,69]. We have further described the different pockets of the selected enzymes in our previous articles [62,70]. Furthermore, the binding affinity between AChE and CID: 107905 was −12.2 kcal/mol, and between AChE and CID: 12000657 it was −11.6 kcal/mol, which was better than the control drug rivastigmine, which has a binding affinity of −7.8 kcal/mol (Table 1; Figure 1).

**Figure 2:**
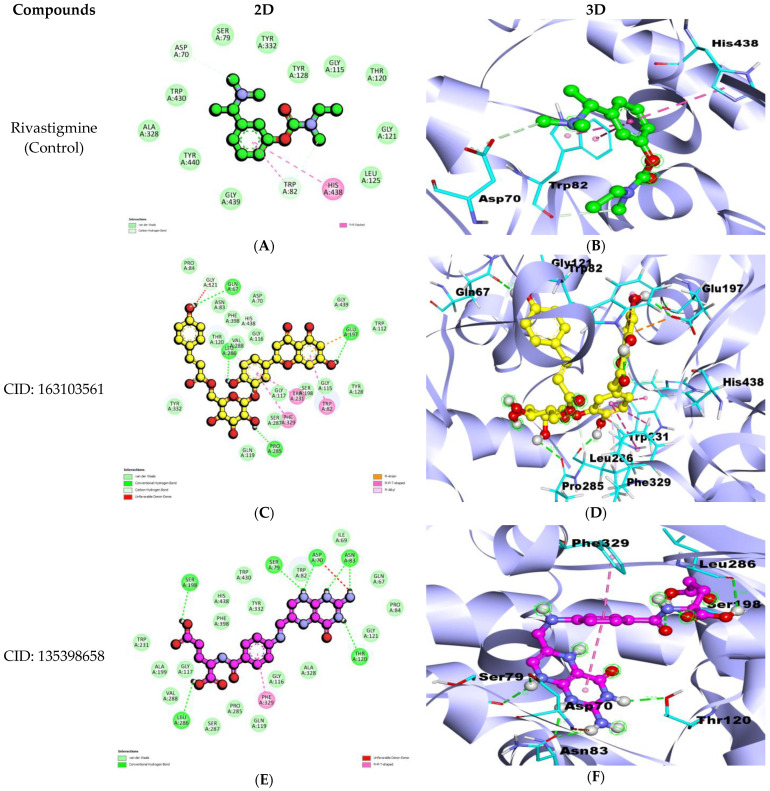
2D representations (**A**,**C**,**E**) and 3D conformations (**B**,**D**,**F**) of interactions of the selected natural compounds with BuChE.

The BuChE and CID: 163103561 interaction showed a binding affinity of −11.2 kcal/mol, while that with CID: 135398658 was −10.0 kcal/mol, better than the control drug rivastigmine (−6.8 kcal/mol) (Table 2; Figure 2).

CID: 163103561 interacted with BuChE with a binding affinity of −11.2 kcal/mol and formed seven hydrogen bonds. Amino acid residues Pro84, Tyr332, Gln119, Asn83, Phe398, Val288, Gly116, Gly117, Ser287, Ser198, Gly115, Gly439, and Trp112 were involved in the Van Der Waals interactions. Also, Glu197 formed a Pi–Anion bond, Leu286 formed a Pi–Alkyl bond, while Phe329, Trp231, and Trp82 formed Pi–Pi T-shaped bonds (Table 2; Figure 2C,D).

The BuChE interaction with CID: 135398658 has shown a −10.0 kcal/mol binding affinity and formed seven hydrogen bonds. Amino acid residues Trp231, Ala199, Val288, Ser287, Pro285, Gln119, Gly116, Ala328, Phe398, His438, Tyr332, Trp430, Trp82, Ile69, Gln67, Pro84, Gly121, and Thr120 were involved in Van Der Waals interactions, while Phe329 formed a Pi–Pi T-shaped bond (Table 2; Figure 2E,F).

Furthermore, active site interaction investigation revealed that CID: 107905 formed a hydrogen bond with Ser203, which is an essential residue of the catalytic triad site of AChE; Trp86, which is a key amino acid residue of the choline-binding site, and Trp286, a component of the peripheral anionic pocket, created a Pi–Pi interaction (Table 1; Figure 1C) [70,71]. Also, it was observed that CID: 12000657 interacted with Asp74 and forming hydrogen bonds. Asp74 facilitates ligand binding with AChE [72]. Choline binding residue Trp86 and acyl pocket residue Phe295 were involved in hydrophobic interactions, while another acyl pocket residue, Phe297, was involved in Pi–Pi bonding (Table 1; Figure 1E). BuChE interaction with CID: 163103561 showed the formation of hydrogen bonds with Leu286, a part of the acyl pocket of BuChE, and with His438, a residue of the catalytic triad. Another essential amino acid residue of the peripheral anionic pocket, Trp82, formed a Pi–Pi T-shaped bond. An aryl residue, Trp332, formed a hydrophobic interaction, and Phe329 formed a Pi–Pi T-shaped bond (Table 2; Figure 2C). During CID: 135398658 interaction with BuChE, Asp70 formed hydrogen bonds, part of the peripheral anionic pocket that enables the compound binding with BuChE. Aryl residue Phe329 formed a Pi–Pi T-shaped bond, and these residues pull compounds toward the deep gorge (Table 2; Figure 2C) [68,69,70].

### 3.2. Drug-Likeness and ADMET Analysis

Based on the ADME data obtained from the SwissADME server, after analyzing several parameters like gastrointestinal (GI) absorption, blood–brain barrier (BBB) permeability, P-glycoprotein substrate interaction, cytochrome inhibition, and log Kp value for skin permeation, showed better results for all the selected compounds. In contrast, compounds 163103561, 12000657, and 107905 showed GI absorption. Compounds 12000657 and 107905 showed CYP1A2 inhibitor properties, and compound 107905 can also inhibit CYP2D6 and CYP3A4 (Table S1; Supplementary File). Drug-likeness analysis revealed that compounds 163103561 and 107905 have zero violation of the required parameters of Lipinski’s rule of five [73] (Table S2; Supplementary File). Further, the toxicity analysis performed using the pkCSM server (http://biosig.unimelb.edu.au/pkcsm/theory) (accessed on 14 January 2023) [61] suggested that all the selected compounds are non-toxic. Compounds fulfilled the criteria set up by different parameters like AMES toxicity, hepatotoxicity, *T. pyriformis* toxicity, and Minnow toxicity, except compound 12000657, which can produce skin sensitization (Table S3, Supplementary File).

### 3.3. MDS Results

**Figure 3:**
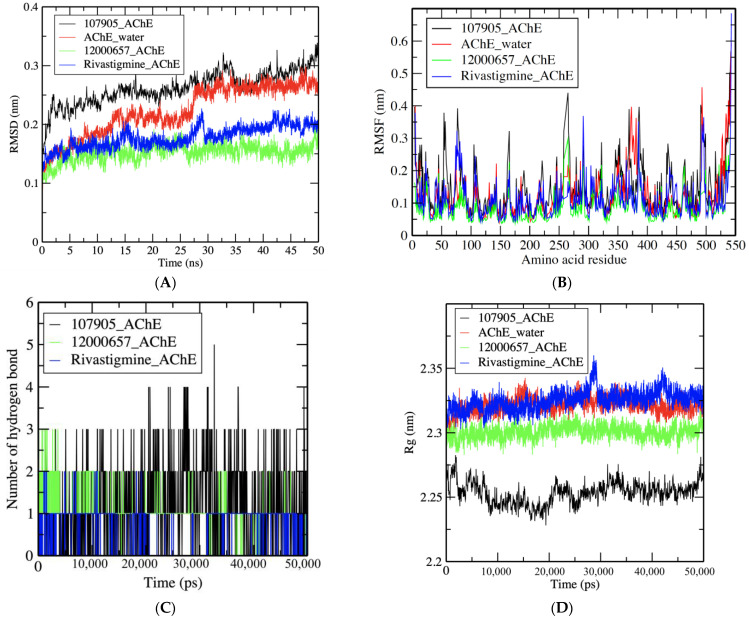
(**A**) RMSD plot of 107905–AChE (black), AChE in water (red), 12000657–AChE (green), and control drug rivastigmine–AChE (blue) complexes. (**B**) RMSF values of the enzyme and compound complexes per amino acid residue. (**C**) Number of hydrogen bonds formed between compounds and AChE in 50,000 ps. (**D**) Rg values that show the compactness of AChE and compounds complexes maintained for the whole simulation period.

RMSD, RMSF, the radius of gyration, and the formation of hydrogen bond plot data were extracted from trajectory files after a 50 ns molecular dynamics simulation. The deviation of all the selected complexes and the AChE simulation in water ranged from 0.1 to 0.3 nm (Figure 3A). The 12000657–AChE complex demonstrated a better and lower RMSD value than the control drug–AChE complex, i.e., near 0.15 nm. It also had the lowest AChE simulation value in water.

RMSF fluctuation plot values ranged between 0.1 and 0.6 nm (Figure 3B) for complexes. In comparison, the observed average value was approximately 0.1 to 0.15 nm, except for some major fluctuations at the 50–60, 70–80, 110–130, 160–170, 260–290, and the 360 amino acid residue regions.

Because of the presence of natural compounds, the radius of gyration analysis is critical for assessing the compactness and stability of protein structures throughout the simulation period. Rg values were observed to be between 2.25 and 2.35 nm. Surprisingly, the complex 12000657–AChE showed promising results compared to the control drug regarding stability, with an average value of 2.3 nm (Figure 3C). While the values for compound–AChE in water and the control drug complex were similar, slightly greater than 2.3 nm, 1–6 hydrogen bonds formed during the 50 ns MDS (Figure 3D). Hydrogen bonds were formed in the 12000657–AChE complex, the rivastigmine–AChE complex, and the 107905–AChE complex.

**Figure 4:**
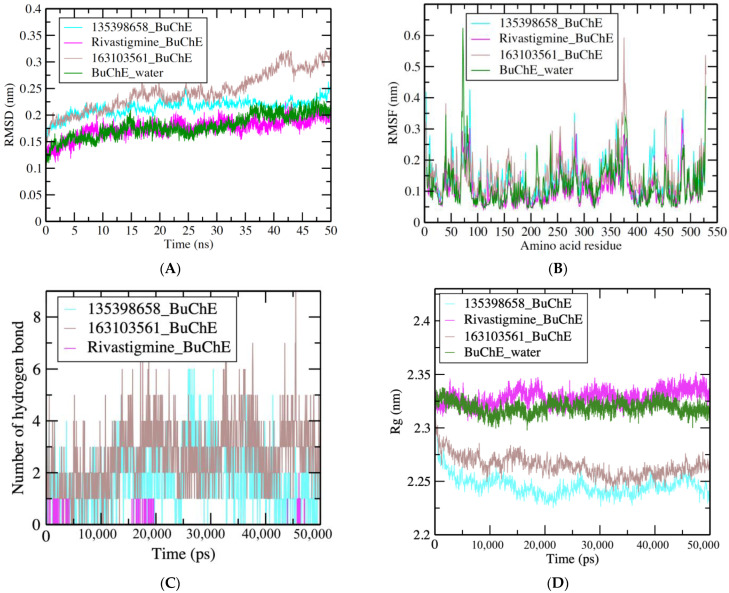
(**A**) RMSD plot of 135398658–BuChE (turquoise), control drug rivastigmine–BuChE (pink), 163103561–BuChE (brown), and BuChE in water (dark green) complexes. (**B**) RMSF values of the enzyme and compound complexes per amino acid residues. (**C**) Number of hydrogen bonds formed between compounds and BuChE in 50,000 ps. (**D**) Rg values that show the compactness of BuChE and compounds complexes maintained for the whole simulation period.

Furthermore, the deviation of all the selected complexes and the BuChE simulation in water showed values between 0.1 and 0.25 nm (Figure 4A). It was observed that the 135398658–BuChE complex showed a stable pattern with an RMSD value of 0.2 nm, which is very near to the control drug–BuChE complex, i.e., approximately 0.15 nm, and a similar value was obtained for the BuChE simulation in water. RMSF fluctuation plot values were between 0.1 and 0.6 nm (Figure 4B) for complexes, while the observed average value was approx. under 0.1 nm, except for some major fluctuations at the 55–75, 350–385, 450–460, and 475–490 amino acid residue regions. The observed values of Rg were between 2.25 and 2.35 nm. The complexes 135398658–BuChE and 163103561–BuChE showed less value in water than the control drug and BuChE, i.e., between 2.25 and 2.3 nm (Figure 4C). During the 50 ns MDS, 1–8 hydrogen bonds were formed (Figure 4D). The compound 135398658–BuChE complex formed 1–6 hydrogen bonds, the rivastigmine–AChE complex formed 1–2 hydrogen bonds, and the compound 163103561–BuChE complex formed 1–8 hydrogen bonds (Figure 4D).

CID: 107905 ((-)-Epicatechin gallate) is a polyphenol that interacted significantly with AChE. A study conducted in 2021 investigated and concluded that exosomes delivered Epicatechin gallate into SHSY5Y cells and demonstrated neuroprotective effects in vitro in a rotenone (Rot)-induced Parkinson’s disease (PD) model [74]. Another study reported that polyphenol Epigallocatechin-3-gallate (EGCG) showed protective effects by reducing neuroinflammation and mitigating neural damage [75]. CID: 12000657 has revealed that the second highest binding affinity is a cysteine proteinase inhibitor purified from the apple fruit [76]. Previous studies suggested that reversible cysteine protease inhibitors have significant properties and could be established as agents for treating AD and other neurodegenerative disorders [77,78]. The identified compound CID: 163103561, which interacts well with BuChE, is a natural product found in *Malus pumila* and *Malus domestica*, with data available [79]. CID: 163103561 was found in the young leaves of *Malus domestica* after treatment with prohexadione-Ca, which is used to reduce the effect of fire blight caused by *Erwinia amylovora*.

Another compound that interacted and showed a better binding affinity with BuChE was CID: 135398658, known as folic acid. As a dietary supplement, apple vinegar benefits anemia patients because it has iron, vitamin B12, and folic acid. Also, apple cider vinegar could have a beneficial effect on asthma, kidney stones, arthritis, and skin diseases patients [80,81,82]. Furthermore, previous studies suggest that diets containing folic acid can prevent neurological disorders, neural tube defects, development delays, and Alzheimer’s disease [83,84,85]. Folic acid, in combination with vitamin B12, could have important preventive functions for CNS developmental and mood disorders, including dementia in Alzheimer’s disease and vascular dementia in older adults [86].

## 4. Conclusions

AChE and BuChE are two types of cholinesterases found in the brain that are associated with choline metabolism. Activation of AChE rapidly hydrolyzes acetylcholine, halting impulse transmission at cholinergic synapses. Cholinesterase inhibitors play a role in various neurodegenerative diseases, including Alzheimer’s. For this reason, neuroscientists have been motivated to seek out and utilize the many naturally occurring compounds in plants worldwide that can inhibit AChE and BuChE. The results of this study indicate that some of the apple’s chemical constituents interact significantly with the enzyme acetylcholinesterase and could be used to improve the health and well-being of those who suffer from neurological diseases. Our research concludes that some of the apple’s natural compounds could be potential treatments for neurological disorders including Alzheimer’s disease.

## Figures and Tables

**Table 1:** Molecular docking data was obtained from the PyRx tool after performing molecular interactions between the selected natural compounds and AChE (PDB:7E3H).

Compounds	Binding Affinity (Kcal/mol)	Hydrogen Bond Names	Hydrogen Bond Lengths (Angstrom)	Van Der Waals Interactions	Other Types of Bond Formation
Rivastigmine (Control)	−7.8	A:TYR337:HH-UNL1:O1	2.15667	GLY120, GLY121, SER203, HIS447, TYR133, GLY448, TRP439, SER125,	PI–PI TYR124 PI–ALKYL TYR337 PI–SIGMA TRP86
		A:TRP86:CD1-:UNL1:O1	3.46816		
		:UNL1:C3-A:THR83:O	3.0719		
		:UNL1:C3-A:ASN87:OD1	3.32779		
		:UNL1:C13-A:GLU202:OE1	3.47924		
(-)-Epicatechin gallate CID: 107905	−12.2	A:TYR337:HH-N:UNK1:O	2.57161	ALA204, GLY122, HIS447, THR83, ASP74, GLY120, GLY121, PRO88, LEU130, GLY126, VAL294, TYR72, PHE295, PHE297, PHE338	PI–PI TRP286, TYR341
		N:UNK1:H-A:TRP86:O	2.56415		
		N:UNK1:H-A:ASN87:OD1	2.57352		
		N:UNK1:H-A:SER203:OG	1.92587		
		A:SER125:HB1-N:UNK1:O	2.81437		
		A:TYR124:HH-N:UNK1	2.80141		
4-((4′-(Aminomethyl)-[1,1′-biphenyl]-3-yl)oxy)pyrimidine-2-carbonitrile CID: 12000657	−11.6	A:ASP74:HN-N:UNK1:N	2.56919	TYR72, VAL73, TRP86, ASN87, TYR124, GLY121, SER125, GLY126, TRP286, VAL294, PHE295, ARG296, PHE338	PI–PI PHE297, TYR337, TYR341
		N:UNK1:HN-A:ASP74:OD2	2.10567		
		N:UNK1:HN-A:SER125:OG	2.25043		
		N:UNK1:H-A:TYR341:O	2.58107		
		N:UNK1:H-A:SER293:O	2.6484		
		A:VAL73:HA-N:UNK1:N	3.01081		

**Table 2:** *In silico* docking data was obtained from the PyRx tool after performing molecular interactions between the selected natural compounds and BuChE (PDB:7AIY). In the hydrogen bond names column, where UNL1, UNK1 = selected compounds.

Compounds	Binding Affinity (Kcal/mol)	Hydrogen Bond Names	Hydrogen Bond Lengths (Angstrom)	Van Der Waals Interactions	Other Types of Bond Formation
Rivastigmine (Control)	−6.8	:UNL1:C3-A:TRP82:O	3.42913	ALA328, TRP430, TYR440, GLY439, SER79, TYR332, TYR128, GLY115, THR120, GLY121, LEU125,	PI–PI STACKING HIS438
		:UNL1:C13-A:ASP70:OD1	3.52033		
[(2R,3S,4S,5S,6S)-6-[5-[(2S)-5,7-dihydroxy-4-oxo-2,3-dihydrochromen-2-yl]-2-hydroxyphenoxy]-3,4,5-trihydroxyoxan-2-yl]methyl (E)-3-(4-hydroxyphenyl)prop-2-enoate CID: 163103561	−11.2	N:UNK1:H-A:GLU197:OE1	2.48466	PRO84, TYR332, GLN119, ASN83, PHE398, VAL288, GLY116, GLY117, SER287, SER198, GLY115, GLY439, TRP112	PI–ANINON GLU197 PI–ALKYL LEU286 PI–PI T SHAPED PHE329,TRP231, TRP82
		N:UNK1:H-A:LEU286:O	1.8801		
		N:UNK1:H-A:PRO285:O	2.54319		
		N:UNK1:H-A:GLN67:OE1	2.29008		
		A:GLY121:HA1-N:UNK1:O	2.84111		
		A:LEU286:HA-N:UNK1:O	2.85756		
		N:UNK1:C-A:HIS438:NE2	3.13846		
Folic acid CID: 135398658	−10.0	N:UNK1:HN-A:ASP70:OD1	2.30031	TRP231, ALA199, VAL288, SER287, PRO285, GLN119, GLY116, ALA328, PHE398, HIS438, TYR332, TRP430, TRP82, ILE69, GLN67, PRO84, GLY121, THR120	PI–PI T SHAPED PHE329
		N:UNK1:HN-A:SER79:O	2.58808		
		N:UNK1:HN-A:ASN83:OD1	2.26671		
		N:UNK1:H-A:THR120:OG1	2.52606		
		N:UNK1:H-A:ASN83:OD1	2.12821		
		N:UNK1:H-A:SER198:OG	2.55222		
		N:UNK1:H-A:LEU286:O	2.84045		

## Data Availability

Not applicable.

## Supplementary Materials

The following supporting information can be downloaded at: https://www.mdpi.com/article/10.3390/nu15071579/s1, Figure S1. Showing 3D model of AChE binding pocket of selected natural compounds CID:107905 (red), CID: 12000657 (purple) and Rivastigmine (green); Table S1. ADME prediction from SwissADME (GI = Gastro intestinal, BBB = Blood Brain Barrier, Pgp = P glycoprotein, CYP = Cytochrome, log Kp = skin permeation); Table S2. Drug-likeness prediction from SwissADME server (MW = Molecular Weight, TPSA = total polar surface area, Consensus Log P = average of all predicted Log Po/w; Table S3: Toxicity prediction. Data obtained from the pkCSM server (http://biosig.unimelb.edu.au/pkcsm/theory) (accessed on 14 January 2023); Table S4: Selected apple natural compounds 2D structure and corresponding binding affinity Data obtained from docking analysis.
