# Supplementary material for: Identification of Natural Compounds of the Apple as Inhibitors against Cholinesterase for the Treatment of Alzheimer’s Disease: An In Silico Molecular Docking Simulation and ADMET Study
Source: Nutrients. 2023 Mar 24;15(7):1579. doi: 10.3390/nu15071579 (PMC10097405; doi:10.3390/nu15071579)
Supplement: Supplementary file 1 [file nutrients-15-01579-s001.zip › nutrients-2261594-supplementary.pdf]

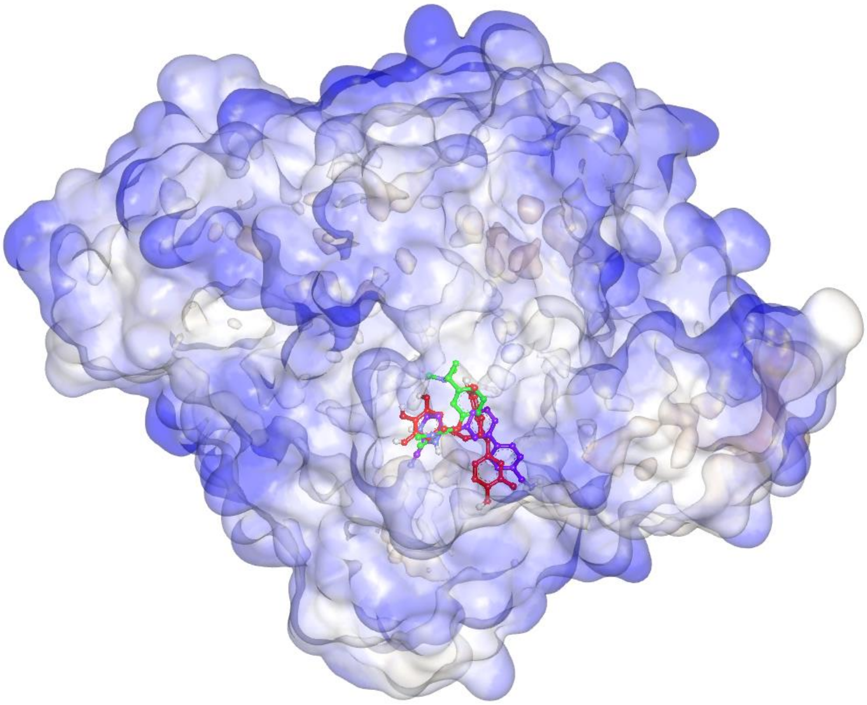

Figure S1. Showing 3D model of AChE binding pocket of selected natural compounds CID:107905 (red), CID: 12000657 (purple) and Rivastigmine (green)

TableS1. ADME prediction from SwissADME (GI=Gastro intestinal, BBB=Blood Brain Barrier, Pgp=P glycoprotein, CYP=Cytochrome, log Kp= skin permeation)									
**Compounds**	**GI absorption**	**BBB permeant**	**Pgp substrate**	**CYP1A2 inhibitor**	**CYP2C19 inhibitor**	**CYP2C9 inhibitor**	**CYP2D6 inhibitor**	**CYP3A4 inhibitor**	**log Kp (cm/s)**
									negative the log *K*p the less skin permeant the molecule
163103561	High	No	No	No	No	No	No	No	-6.77
135398658	Low	No	No	No	No	No	No	No	-8.42
12000657	High	No	No	Yes	No	No	No	No	-2.24
107905	High	No	No	Yes	No	No	Yes	Yes	-6.9

Table S2. Drug-likeness prediction from SwissADME server (MW=Molecular Weight, TPSA= total polar surface area, Consensus Log P= average of all predicted Log Po/w

Compounds	MW (g/mol)	Rotatable bonds	H-bond acceptors	H-bond donors	TPSA (Å²)	Consensus Log P	Lipinski violations	Ghose violations	Veber violations	Egan violations	Muegge violations	Bioavailability Score	Synthetic Accessibility
													normalized between 1 (easy synthesis) and 10 (very difficult synthesis)
163103561	198.17	3	5	2	75.99	0.99	0	0	0	0	1	0.56	1.7
135398658	448.38	3	11	7	190.28	0.19	2	0	1	1	3	0.17	5.28
12000657	298.5	17	2	0	26.3	6.24	1	1	1	1	2	0.55	2.95
107905	316.26	2	7	4	120.36	1.65	0	0	0	0	0	0.55	3.26

Table S3: toxicity prediction. Data obtained from the pkCSM server (http://biosig.unimelb.edu.au/pkcsm/theory **)**

	AMES toxicity	Max. tolerated dose (Human)	hERG I inhibitor	hERG II inhibitor	Oral Rat Acute Toxicity (LD50)	Oral Rat Chronic Toxicity (LOAEL)	Hepatotoxicity	Skin sensitisation	T. pyriformis toxicity	Minnow toxicity
**Standard cut-off**	**Positive mutagenic**	**<= 0.477 log(mg/kg/day)**				**lowest**			**> -0.5 log ug/L**	**Log LC50< -0.3**
**Compounds**										
163103561	No	1.374	No	No	2.157	2.415	No	No	0.281	2.554
135398658	No	0.495	No	No	2.586	3.022	No	No	0.285	4.954
12000657	No	0.297	No	No	1.601	3.099	No	Yes	1.719	-1.751
107905	No	0.576	No	No	2.407	2.499	No	No	0.296	2.206

Table S4: Selected apple natural compounds 2D structure and corresponding binding affinity Data obtained from docking analysis

Compound IDs	2D pictures	Binding affinity (Kcal/mol)
CID: 107905	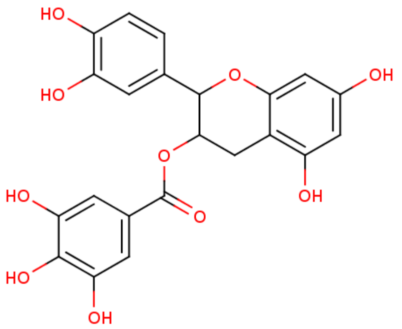	-12.2
CID: 12000657	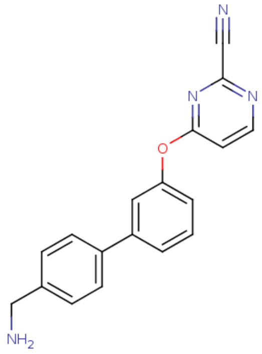	-11.6
CID: 163103561	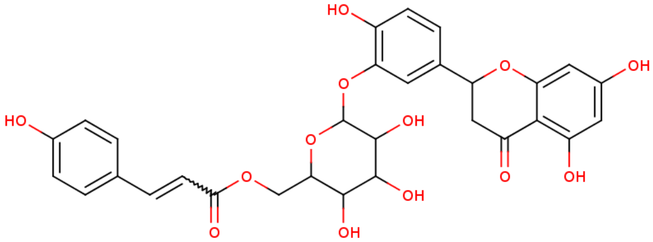	-11.2

CID: 135398658	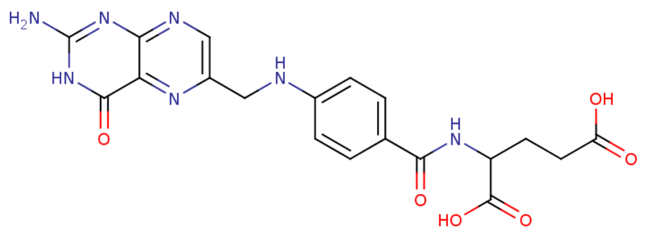	-10.0
